# Fluorescent hydrogel waveguide for on-site detection of heavy metal ions

**DOI:** 10.1038/s41598-017-08353-8

**Published:** 2017-08-11

**Authors:** Jingjing Guo, Minjuan Zhou, Changxi Yang

**Affiliations:** State Key Laboratory of Precision Measurement Technology and Instruments, Department of Precision Instruments, Tsinghua University, Beijing, 100084 China

## Abstract

Hydrogels have great applications in tissue engineering and drug delivery. Recently, there have been intense interests in developments and applications of nanocomposite hydrogels by incorporating nanomaterials into polymer matrix, which endows the hydrogels with new functionalities. Here, we report on the first carbon dots (CDs) doped hydrogel waveguide for selective, on-site detection of heavy metal ions in aqueous solutions. The CDs-doped hydrogel waveguide exhibits efficient light confinement in water due to the refractive index contrast. The smooth waveguide surfaces lead to low light scattering loss. Real-time spectra measurement of the CDs-doped hydrogel waveguide with a compact interrogation setup demonstrates that the novel design can be used as a portable, robust sensing platform for on-site analysis and assessment of heavy metal ions.

## Introduction

Environmental contamination by heavy metals has been a serious threat to the ecological system and global public health due to their increasing use in industrial, agricultural, and medical applications^1, 2^. Mercury, for instance, can induce severe damage to the central nervous system and kidney failure even at a low exposure^3^. Various methods, such as atomic fluorescence spectrometry^4^, atomic absorption/emission spectroscopy^5^, and inductively coupled plasma mass spectrometry^6^, have been traditionally employed for quantitative detection of heavy metal ions with high sensitivity. These techniques, however, normally require complicated instrumental settings and chemical processes to extract metal ions from water samples, which are not feasible for rapid on-site detection^7^.

Advances in fluorescent nanomaterials have paved the way of exploring new tools for bioimaging and biosensing^8–11^. Recently, carbon dots (CDs) as an emerging new class of fluorescent nanoparticles have attracted significant interests owing to their robust chemical inertness, low cytotoxicity, and good biocompatibility compared with conventional organic dyes and semiconductor quantum dots^12, 13^. In addition to their extensive applications in photocatalyst and bioimaging^14, 15^, CDs have been demonstrated as a promising fluorescent sensor for detection of heavy metal ions such as Fe^3+^, Hg^2+^, Pb^2+^, Cd ^2+^, Cu^2+^, *etc*.^16–20^. However, CDs as sensing probes typically need to be mixed with the target aqueous samples, which makes the CDs-based probes not applicable for on-site detection. Moreover, the fluorescence intensity of CDs can be affected by a number of analyte-independent aspects, such as the light scattering by the sample matrix, probe concentration, and environmental conditions, which cause difficulties in precise and repeatable measurements. To overcome such drawbacks, much effort has been devoted to immobilizing CDs on surface of silica optical fibers, where the CDs were excited by evanescent wave^21, 22^. However, the weak light coupling efficiency with evanescent wave and the fragile nature of the silica fiber tip pose challenges for their on-site detection.

The design of various hydrogels as light-guiding platform integrated with photonic functions has aroused great interests for sensing and therapy applications^23–25^. The porous matrix of the hydrogels allows small molecules and ions penetrate into the hydrogels via diffusion. Functional molecules comparable or larger than the pore size can be physically immobilized in the matrix^26, 27^. Immobilization of the CDs in a hydrogel matrix provides the possibility of on-site detection of the heavy metal ions and minimizes the analyte-independent effects caused by sample conditions^28–33^. A common approach in previous reports was dipping the CDs-incorporated hydrogel into the aqueous sample and then taking it out for optical detections^28, 30, 32^. Unfortunately, these employments for detection of metal ions have not yet achieved real-time *in-situ* operation due to the limitation of laser delivery and emission collection in aqueous environments.

In this study, we demonstrate the design and fabrication of Hg^2+^-sensing optical waveguide made of CDs incorporated hydrogels. The CDs-doped hydrogel waveguide has a high quality of light confinement in aqueous solution due to high refractive index contrast between the hydrogel and water. The smooth hydrogel waveguide surfaces lead to low light scattering loss. The hydrogel waveguide pigtailed with standard optical fiber enables on-site detection of heavy metal ions in real time. Systematic characterization and optimization of the materials were performed to facilitate waveguide fabrication with desired functionalities. We show that the hydrogel waveguide can achieve efficient laser excitation and emission collection in aqueous samples due to light confinement within the optical device, meanwhile heavy metal ions can penetrate into the porous matrix by diffusion and interact with the entrapped functional CDs. The waveguide was interrogated by a compact all-optical fiber setup, which makes it also possible for remote sensing due to the low propagation loss of optical fibers. Quantitative characterization and real-time optical measurement of the waveguide in aqueous solutions shows that the fabricated waveguide can be used as a portable, robust sensing platform for rapid, selective and on-site detection of Hg^2+^ ions.

## Results and Discussion

### Characteristics of the hydrogels

**Figure 1:**
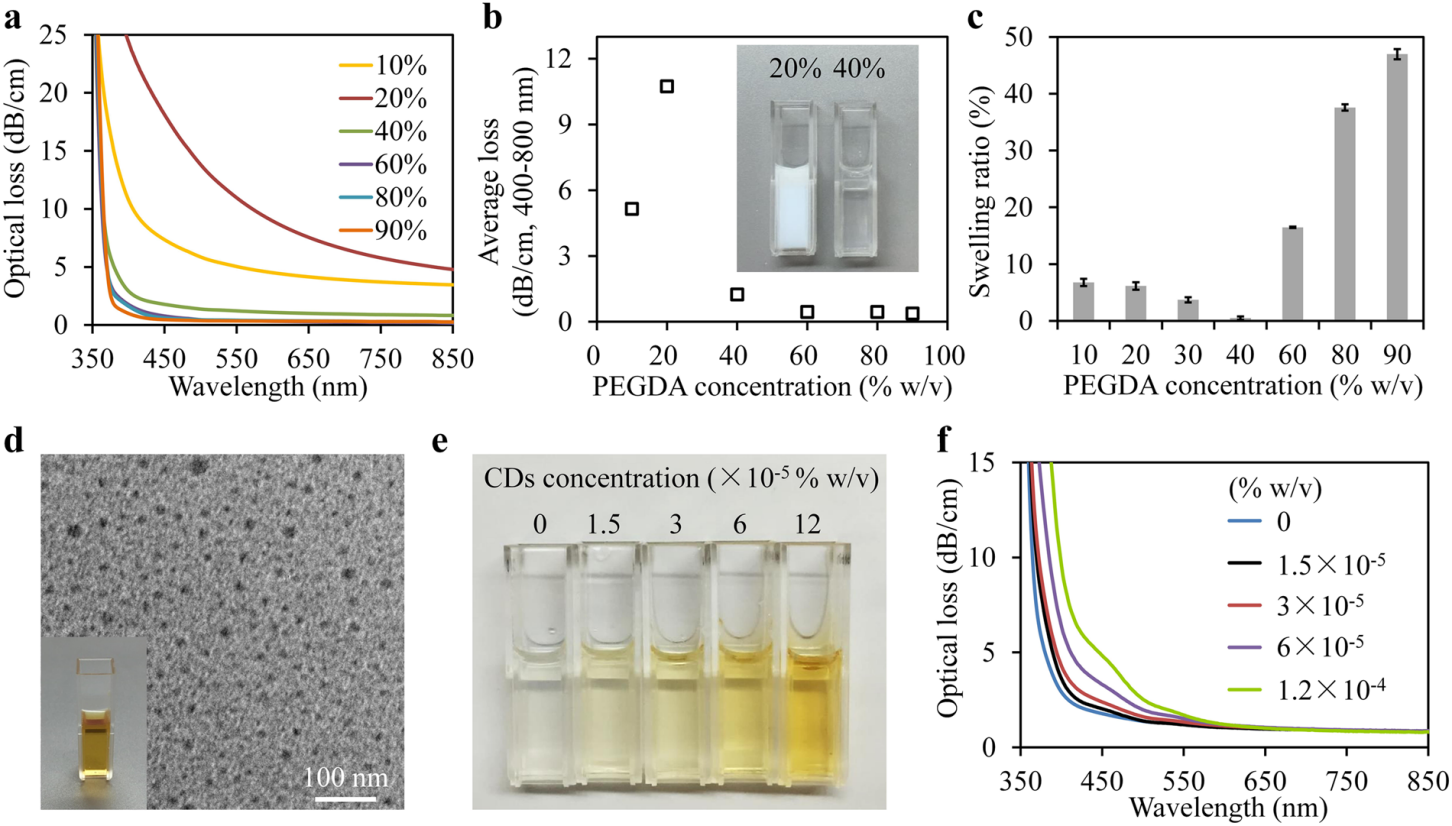
(**a**) Optical loss spectra of PEGDA hydrogels at concentrations from 10% to 90% w/v. (**b**) Dependence of the optical loss of hydrogels on PEGDA concentration, averaged in spectrum range of 400–800 nm. The inset shows the photograph of PEGDA Hydrogels at concentration of 20% and 40%. (**c**) Swelling ratio of the PEGDA hydrogels in deionized water at 25 °C. The swelling ratio was determined from the measured weight ratio between the fully swollen and as-made hydrogel. Error bars, standard deviations (n = 3). (**d**) TEM image of CDs. (**e**) CDs-PEGDA nanocomposites with PEGDA concentration of 40%w/v and CDs concentrations from 0 to 1.2 × 10^−4^% w/v. (**f**) Optical loss spectra of CDs-PEGDA nanocomposites at various CDs concentrations.

We chose the widely used biocompatible hydrogels: PEG diacrylate (PEGDA) to make the slab waveguide. To determine the optimal PEGDA concentration for the waveguide fabrication, we characterized the optical and swelling properties of the hydrogel. The optical loss spectra of PEGDA hydrogels with various concentrations were measured in standard 1-cm-wide cuvettes. The transparency of the hydrogel was found strongly dependent on the PEGDA concentration attributed to the phase separation between the water-rich phase and the polymer-rich phase (Fig. 1a)^27, 34^. At PEGDA concentrations of 10–20%, the hydrogels were white opaque and indicated significant loss higher than 5.15 dB/cm (Fig. 1b). For PEGDA concentrations at or above 40%, the hydrogels became remarkably transparent and the average loss in range of 400–800 nm was less than 1.25 dB/cm. Swelling test was also performed by immersing the hydrogels in deionized water for 24 hours. The hydrogels were found seriously deformed and even cracked at PEGDA concentration higher than 60% due to large swelling whereas negligible swelling effect was observed at 40% (Fig. 1c). Considering both the optical transparency and swelling properties, we chose 40% PEGDA for the waveguide fabrication.

### Carbon dots incorporated nanocomposite hydrogels

The pore size of PEGDA hydrogels with molecular weight of 700 Da is less than 2 nm^35^. Fluorescent carbon dots were synthesized from citric acid by hydrothermal method (Fig. 1d) and have shown high selectivity of fluorescence quenching toward Hg^2+^ ions in previous studies attributed to non-radiative electron transfer^36, 37^. The prepared CDs with an average diameter of about 7.8 nm exhibit peak absorption at around 352 nm and fluorescence emission at 475 nm in water (Figure S1, see supplementary information). CDs with various concentrations could be incorporated into the hydrogel matrix through solution doping prior to photo-crosslinking (Fig. 1e). The resulting CDs-PEGDA nanocomposite shows no changes in optical attenuation at wavelength longer than 550 nm, compared with the PEGDA hydrogel (Fig. 1f). In the wavelength range of 350–550 nm, increased optical attenuation was observed with increasing CDs concentration contributed by the absorption of CDs, which was linearly proportional to the CDs concentration, as expected from Beer-Lambert law (Figure S2).

### Slab CDs-PEGDA waveguides

**Figure 2:**
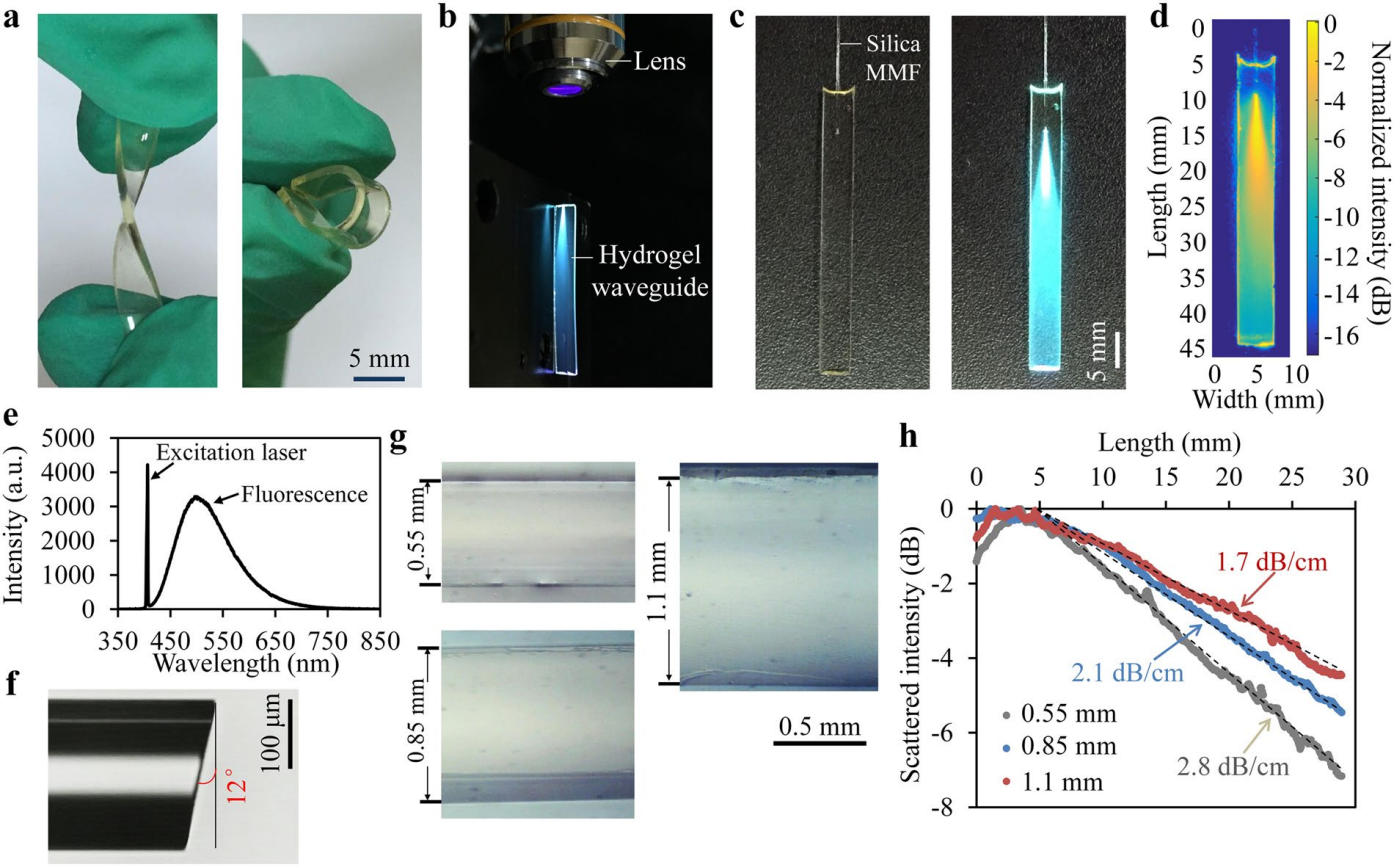
(**a**) Photos of fabricated hydrogel waveguides. (**b**) Light guidance in air. Excitation laser at 405 nm was coupled to a waveguide through an object lens (NA = 0.25). (**c**) Light coupling through a pigtailed silica multimode fiber (MMF). Left, laser off; right, laser on; (**d**) Intensity profile of the scattered light. (**e**) Spectrum of the side-scattered light. (**f**) The coupled silica fiber end cleaved at 12° to suppress the back reflection. (**g**) Optical microscope images showing waveguides thickness at 0.55 mm, 0.85 mm, and 1.1 mm. (**h**) Light guiding losses measured from side-scattered light profiles of the waveguides.

Slab optical waveguides were fabricated by using custom-made rectangular glass molds, where precursor solutions containing PEGDA, carbon dots and photoinitiator were injected through a syringe and polymerized under UV light irradiation (Figure S3). After photo-crosslinking, the waveguides were taken out of the molds by removing the cover slides. The typical dimensions of the fabricated waveguides were 0.55–1.1 mm (thickness) × 5 mm (width) × 40 mm (length). The waveguides could be easily twisted and bent, demonstrating high flexibility and elasticity (Fig. 2a). Light coupling can be realized by launching the excitation violet laser (λ = 405 nm) into the waveguide through an objective lens (Fig. 2b). Alternatively, a silica multimode fiber (MMF) was pigtailed to the waveguide by embedding a short section (~5 mm) of the fiber in the waveguide precursor and the following photo-crosslinking led to a smooth and low-loss connection (Fig. 2c). The fiber-coupling configuration made the device competent for portable, robust and remote operation in practice. The scattering pattern along the waveguide was imaged in air, showing dispersed intensity profile in the waveguide (Fig. 2d). The measured spectrum of the scattered light indicated fluorescence emission at about 485 nm with 10-nm red shift compared with the CDs in DI water and the strong peak at 405 nm was induced by the excitation laser (Fig. 2e). The numerical aperture (NA) of the pigtailed MMF was 0.2, corresponding to an acceptance angle of ~11.5°. To suppress back reflection, the fiber end was cleaved to be 12° (Fig. 2f).

Using molds of different inner thicknesses, waveguides with various thicknesses were fabricated (Fig. 2g). The propagation losses of the waveguides were measured from the intensity profile of side-scattered light by integrating the intensity at the width direction (Fig. 2h). The scattered intensity decreased exponentially (linearly in dB scale) with the propagation length, attributed to material absorption and scattering induced by spatial inhomogeneity. For waveguide with a thickness of 1.1 mm, the loss coefficient was 1.7 dB/cm, but it increased to 2.1 dB/cm and 2.8 dB/cm for waveguide thicknesses of 0.85 mm and 0.55 mm, respectively.

### Excitation and emission collection with a compact fiber-optic setup

**Figure 3:**
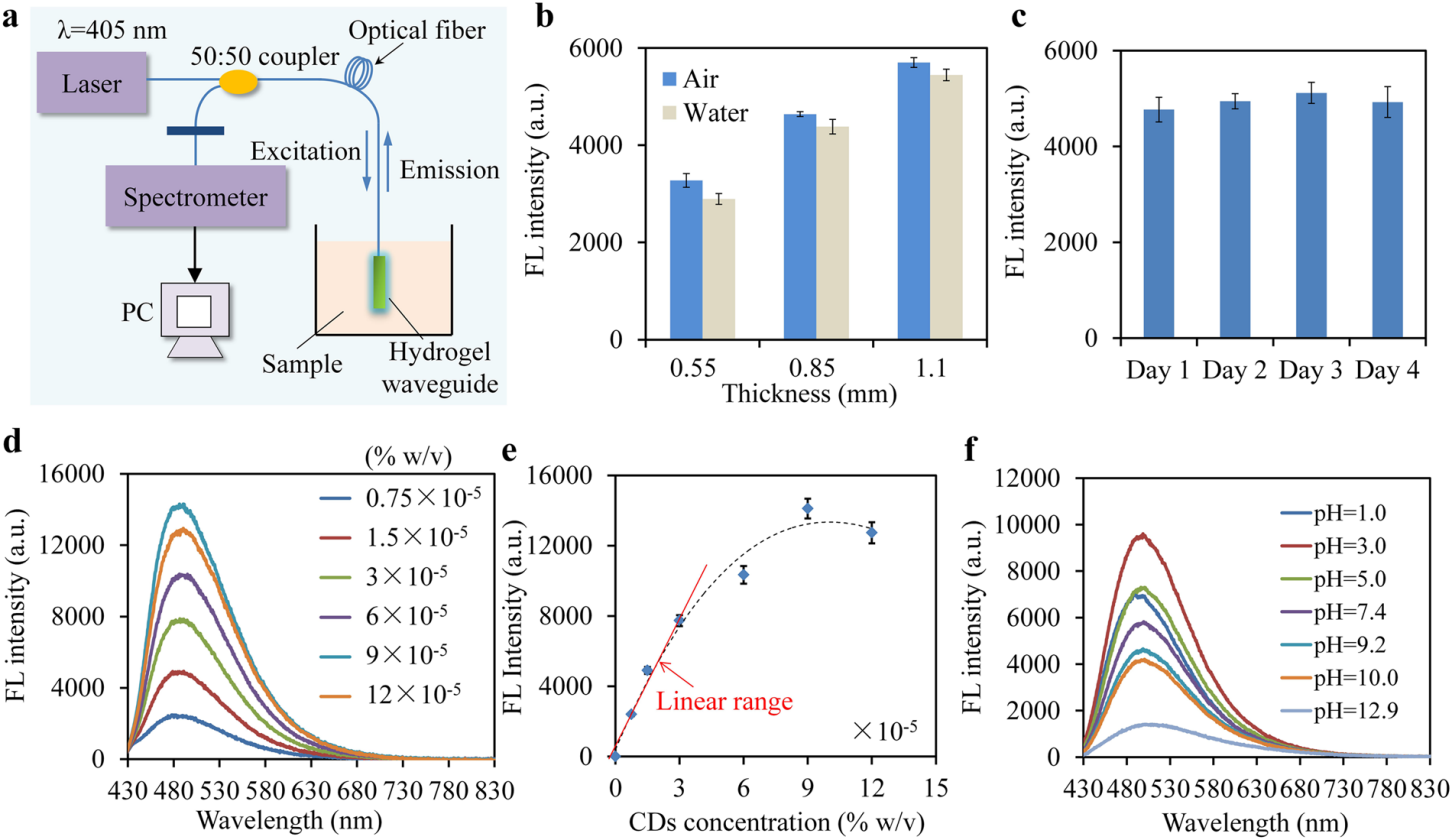
(**a**) Optical setup for excitation and emission collection. (**b**) Effect of the waveguide thicknesses on fluorescence intensity, measured through the pigtailed fiber. (**c**) Long-term stability of fluorescence intensity in DI water. (**d**) Fluorescence spectra of waveguides at CDs concentration of 0.75–12 × 10^−5^% w/v. **(e)** Fluorescence intensity at peak wavelength versus CDs concentration. (**f**) Fluorescence spectra of waveguides in aqueous solutions at pH from 1.0 to 12.9. Error bars, standard deviations (n = 3) in **b, c** and **e**.

We investigated waveguides with dimensions of 1.1 × 5 × 40 mm^3^ at loss coefficient of 1.7 dB/cm. Fluorescence emission from the waveguide was collected to a spectrometer via a fiber coupler (50:50). A high-pass optical filter with a cut-off wavelength of 410 nm was employed to remove laser reflection (Fig. 3a). As hydrogels are commonly used in wet environments, the waveguides were stored in DI water before testing. Light in the hydrogel waveguide was guided via total internal reflection at the interface between waveguide and the surrounding medium following the Fresnel Equation^38^. The refractive index (RI) of the hydrogel was measured to be 1.402. When immersed in DI water (RI, 1.333), the waveguides exhibited slightly decreased fluorescence intensity compared with the case of in air, which was attributed to the smaller index of refraction difference and weaker light guidance in water (Fig. 3b). Decreasing waveguide thickness resulted in decreased intensity of fluorescence due to increased light scattering at the boundary and more light leakage (Fig. 3b), which agrees with the scattering measurements (Fig. 2h).

Immobilization of the CDs in hydrogel matrixes was confirmed by monitoring the fluorescence. The waveguides in DI water exhibited stable fluorescence intensity in observation for 4 days, indicating no leakage of CDs from the hydrogel matrix (Fig. 3c). The effect of CDs concentration on fluorescence emission was also investigated. The intensity of fluorescence increased linearly with the concentration of CDs at concentrations less than 3 × 10^−5^ % w/v (Fig. 3d). However, at concentrations higher than 3 × 10^−5^ % w/v, the linearity was not satisfied due to significant collisional quenching between the fluorescent CDs^39^ (Fig. 3e). Furthermore, the collected fluorescence was found to be highly sensitive to pH changes over the range of 1.0–12.9 (Fig. 3f). The dependence on pH was presumably attributed to changes of the surface state/molecule state of the embedded CDs. When the surrounding pH was too low/high, molecular groups can be strongly affected^40, 41^. The waveguide showed a maximum fluorescence intensity at pH = 3.0 and decreasing intensity at pH lower/higher than 3.0 (Figure S4). Moreover, the fluorescent waveguide indicated a linear response in pH range of 6.2–10.0, with a good correlation coefficient of 0.9884, which also demonstrated the potential of the waveguide for pH sensing in solvent or tissue (Figure S4).

### Fluorescence-based sensing of Mercury ions

**Figure 4:**
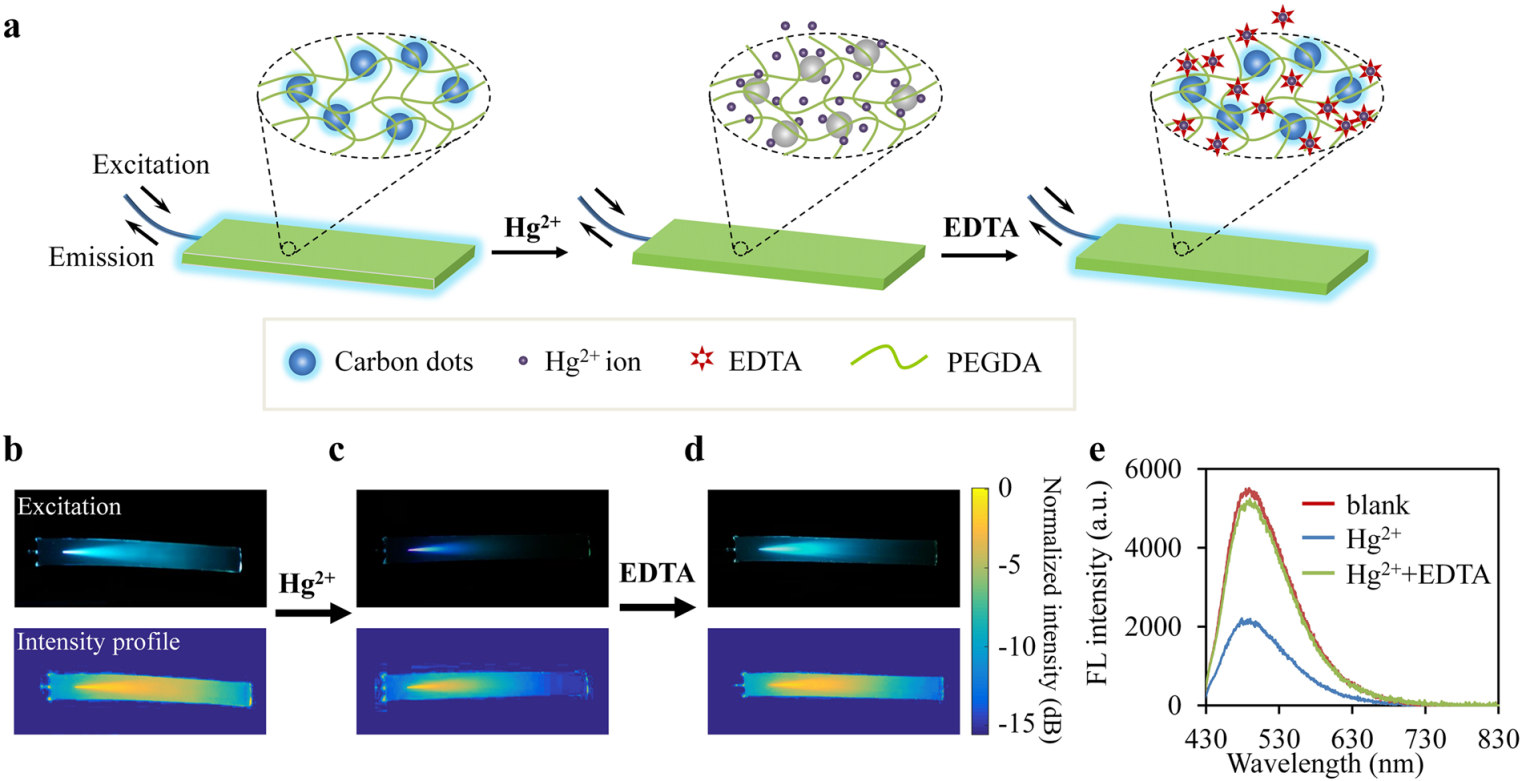
(**a**) Schematic illustration of fluorescence quenching in presence of Hg^2+^ ions and fluorescence recovery with addition of EDTA. (**b**–**d**) Images showing reversible sensing of Hg^2+^. (**e**) Fluorescence spectra measured through the pigtailed fiber.

Hg^2+^ ions are highly toxic and ubiquitously distributed pollutants. Reliable technologies for rapid and on-site monitoring of Hg^2+^ level in aqueous systems have become a critical issue due to its serious threat on human health and the environment. We applied the CDs-PEGDA waveguide for sensing of Hg^2+^ ions by monitoring the fluorescence changes. Fluorescence emission of the CDs results from radiative recombination of the excitons. In the presence of Hg^2+^, fluorescence of the CDs can be quenched attributed to facilitating non-radiative electron transfer from the excited state to the d-orbital of Hg^2+,^ ^3^ (Fig. 4a). With the addition of a strong metal ion chelator such as EDTA, Hg^2+^ is removed from the surface of the CDs and the quenched fluorescence of the CDs can be recovered, allowing reversible response to Hg^2+^ ions. We experimentally validated this mechanism with the fluorescent waveguide. The waveguide was first immersed in aqueous solution with no Hg^2+^ ions and strong fluorescence was observed (Fig. 4b). Then, the waveguide was dipped into Hg^2+^-containing solution and, as expected, the fluorescence was significantly quenched (Fig. 4c). After adding EDTA, the fluorescence intensity and spectrum were basically recovered due to the chelation of Hg^2+^ with EDTA (Fig. 4d and e).

### Selective and quantitative detection of Hg^2+^ ions

**Figure 5:**
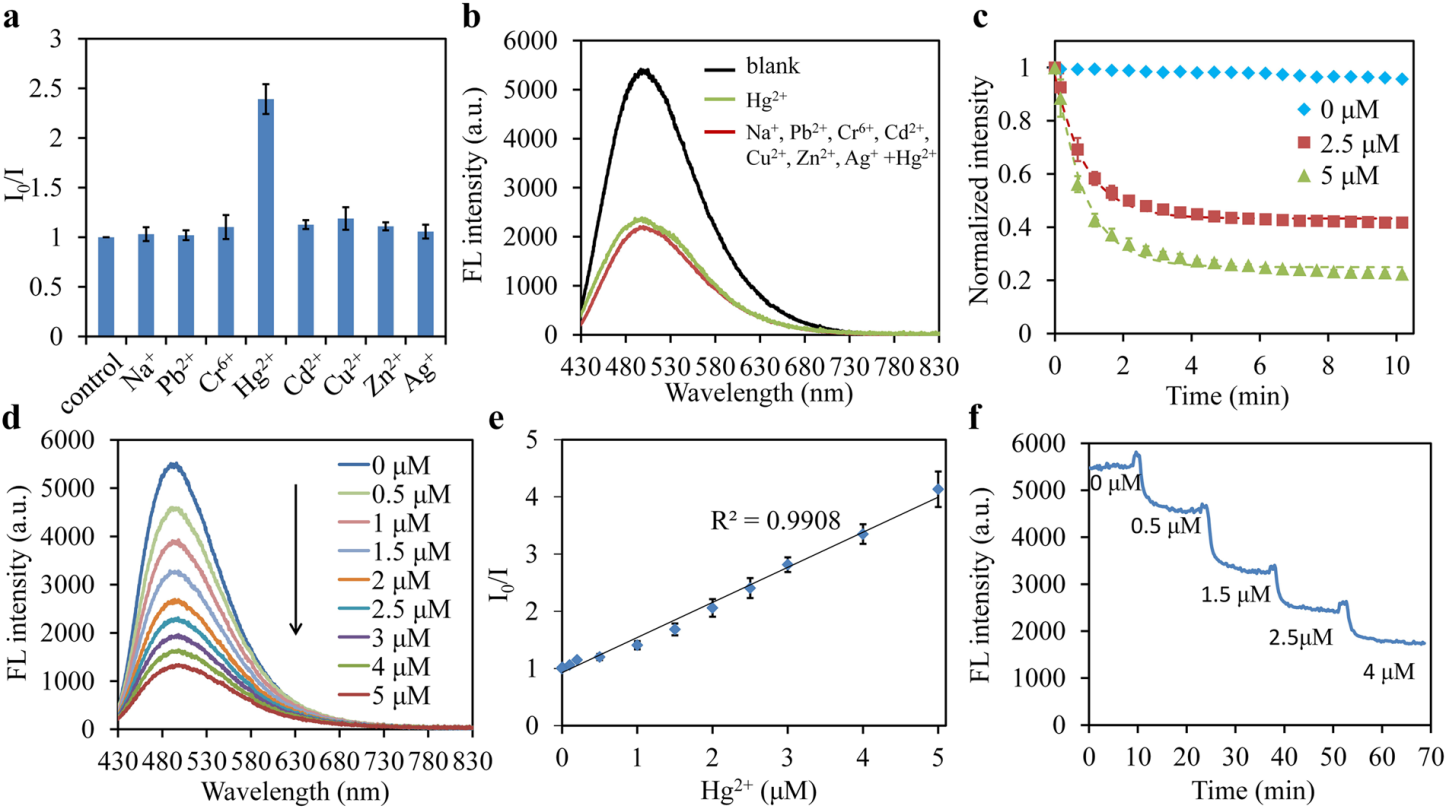
(**a**) Fluorescence response of the waveguides to different metal ions in PBS (pH = 7.4). The concentration of each metal ion was 2.5 μM. (**b**) Fluorescence quenching in presence of coexisting metal ions. **(c)** Time response of fluorescence intensity in absence/presence of Hg^2+^. Dot lines, curve fits with the exponential decay equation. (**d**) Fluorescence spectra at various concentration of Hg^2+^ from 0 to 5 μM. (**e**) Stern-Volmer plot of the fluorescence quenching. (**f**) Real-time readout of fluorescence intensity in tap water with gradually increased concentration of Hg^2+^ ions. Error bars, standard deviations (n = 3) in **a, c** and **e**.

The quenching of a fluorophore results from diffusive collision between the fluorophore and quencher and the quenching process can be described by the Stern-Volmer equation^20^,

1 $$\frac{{I}_{0}}{I}=1+{K}_{SV}[Q]$$

where *I*_0_ and *I* are the fluorescence intensity in the absence and presence of metal ion, *K*_*SV*_ is the Stern-Volmer constant and [*Q*] is the concentration of the metal ion. To study the selectivity of the waveguide towards Hg^2+^ ions, the quenching behavior of the waveguide to various metal ions was measured. The waveguide was dipped into different aqueous solutions containing Na^+^, Pb^2+^, Cr^6+^, Hg^2+^, Cd^2+^, Cu^2+^, Zn^2+^and Ag^2+^ separately. The concentration of each metal ion was kept at 2.5 μM. The fluorescence, measured through the pigtailed fiber was found to be significantly quenched by Hg^2+^ ions, while almost no changes in fluorescence intensity were observed in the presence of other metal ions (Fig. 5a). The quenching coefficient (*I*_0_/*I*) of Hg^2+^ was much higher than that of the others, demonstrating high selectivity of the fluorescent waveguide toward Hg^2+^. The effect of fluorescence quenching in the presence of mixtures of metal ions was also examined. Compared with the effective quenching of Hg^2+^, the other coexisting heavy metal ions showed negligible effects on the fluorescence quenching (Fig. 5b).

The time response of the waveguide to Hg^2+^ was further measured, for which the emission spectra were continuously recorded for 10 min. The waveguide showed stable intensity over time in the absence of Hg^2+^ (Fig. 5c). Once immersed in Hg^2+^-containing solutions, the measured fluorescence decayed rapidly over time attributed to molecular diffusion of the Hg^2+^ ions into the hydrogel matrix and around 5 min later, it reached a relatively stable state implying an optimal time of 5 min for Hg^2+^ detection. The kinetic behavior of the waveguide to Hg^2+^ can be analyzed via an exponential decay model given by:

2 $$F(t)-F(\infty )\propto {e}^{-\alpha t}$$

where *F*(*t*) represents the normalized fluorescence intensity over time, *F*(∞) is the saturated intensity after an infinite time, and *α* is the decay constant, which depicts the affinity of Hg^2+^ with the embedded CDs. This equation fitted well with the measured time-dependency data (Fig. 5c). We probed the potential factors that affected the time response of the waveguide including waveguide thickness and the CDs concentration (Figure S5). The decay constant *α* increased with decreasing waveguide thickness, indicating faster response to Hg^2+^ with thinner waveguide. For waveguides at various concentrations of CDs, the time-dependency trends nearly overlapped in time and the corresponding decay constant was almost independent of CDs concentration.

To evaluate the capability of the waveguide for quantitative detection of Hg^2+^, we dipped the waveguide into solutions with gradually increased concentration of Hg^2+^ from 0 to 5 μM. The waveguide showed decreased fluorescence intensity with increased Hg^2+^ concentration from measurements of the emission spectra (Fig. 5d). The quenching of the fluorescence intensity indicated a linear response (R^2^ = 0.9908) with the Hg^2+^ concentration as expected from equation (1) and the detection limit was estimated to be 4 nM, obtained from three times the standard deviation divided by slope of the calibration curve (Fig. 5e). The excellent selectivity and sensitivity of the waveguide to Hg^2+^ suggest that the photonic device might be directly applied for detection of Hg^2+^ ions in natural water samples. We spiked various concentrations of Hg^2+^ ions with tap water samples, which were obtained from our lab and used without any pretreatment. The sensing waveguide was dipped in samples with gradually increased Hg^2+^ concentration and the fluorescence intensity was found decreased accordingly with fairly fast response (Fig. 5f). Slight increase of the fluorescence intensity was observed each time when the waveguide was taken out and dipped into another sample. This was attributed to the different light guiding efficiency in air and water (Fig. 3b).

## Conclusions

In summary, we demonstrated a portable, robust photonic device for real-time, selective and on-site detection of Hg^2+^ ions based on carbon dots incorporated nanocomposite hydrogels. Functional CDs were entrapped in light-guiding hydrogels and quenched by the Hg^2+^ ions that penetrated into the hydrogel matrix through passive diffusion. Light confinement was achieved via total internal reflection at the interface between the hydrogel and the surrounding aqueous medium. Quantitative characterization of the waveguide for Hg^2+^ sensing indicated a low detection limit of 4 nM and a linear range from 0 to 5 μM. The hydrogel waveguide served as a portable platform, which provided laser delivery, emission collection as well as exchanges with surrounding analytes. Although Hg^2+^ ions were selected as the sensing target in this study, the current design can be applied for sensing of other metal ions by incorporating various CDs functionalized with specific selectivity. The photonic device may provide a promising new approach for on-site analysis and assessment of heavy metal ions in natural environments.

## Materials and Methods

### Slab waveguides made of CDs-PEGDA nanocomposites

Fluorescent carbon dots were synthesized from citric acid with hydrothermal method following previously reported procedures^31, 32^ and the obtained solution was used for waveguide fabrication. To fabricate the fluorescent waveguide, the hydrogel precursor was synthesized by mixing degassed aqueous solution of 40% w/v PEGDA (700 Da, Sigma-Aldrich), cardon dots (0–12 × 10^−5^ % w/v) and 2% w/v 2-hydroxy-2-methyl-propiophenone (Sigma-Aldrich) in deionized water. Afterwards, the precursor was injected in a rectangular glass mold through a syringe and polymerized under UV irradiation (365 nm, 5 mW cm^−2^) for 5 min. The waveguide was then taken out after removing the cover slide of the mold. For light coupling, a silica multimode fiber (core/clad: 200/215 μm) was pigtailed to the waveguide by embedding a short section (~5 mm) of the fiber tip into the precursor and aligned to its center before photo-crosslinking. Additional epoxy resin was used to reinforce the joint. Optical losses of the waveguides at various thicknesses were measured by integrating the intensity at the width direction from the scattering pattern. To measure the refractive index of the carbon dots incorporated hydrogel, 300 μL of the precursor solution were placed on the prism of a digital refractometer (Atago) and the refractive index was measured after photo-crosslinking.

### Instruments and characterization

Transmission electron microscopy (TEM) was conducted using an H-7650B electron microscope (Hitachi). Fluorescence spectra of the CDs excited by laser at 405 nm were recorded by a compact CCD spectrometer (Ocean optics). UV-Vis absorption spectra were measured using a scanning UV-Vis spectrometer (PerkinElmer). Statistical size distribution of the CDs was obtained from 200 CDs. PEGDA hydrogels with various concentrations were prepared in standard 1-cm-wide cuvettes and the optical attenuation of the hydrogels was measured using the UV-Vis spectrometer. Swelling ratio of the hydrogels defined as the weight of fully-swollen hydrogel divided by the as-made hydrogel was measured by immersing the hydrogels in deionized water for 24 hours at 25 °C.

### Optical setup

Excitation laser at 405 nm was coupled to the waveguide through the pigtail fiber and the emission was guided to a spectrometer (Ocean optics) via a 50:50 fiber coupler. A high-pass optical filter with cutoff wavelength of 410 nm was employed to remove laser reflection.

### pH and metal ion titrations

Standard aqueous solutions of Na^+^, Pb^2+^, Cr^6+^, Hg^2+^, Cd^2+^, Cu^2+^, Zn^2+^ and Ag^2+^ in concentration range of 0–5 μM were prepared in PBS (pH = 7.4). For pH measurement, PBS was used for pH from 3.0 to 8.2. For pH below 2.0 and above 9.0, HCl (1 M) and NaOH (1 M) were used to adjust the aqueous solution to the required pH.

## Electronic supplementary material

Supplementary information
