# Supplementary material for: Fluorescent hydrogel waveguide for on-site detection of heavy metal ions
Source: Sci Rep. 2017 Aug 11;7:7902. doi: 10.1038/s41598-017-08353-8 (PMC5554134; doi:10.1038/s41598-017-08353-8)
Supplement: Supplementary file 1 — Supplementary information [file 41598_2017_8353_MOESM1_ESM.doc]

**Supplementary Information**

Fluorescent hydrogel waveguide for on-site detection of heavy metal ions

**Jingjing Guo^†^, Minjuan Zhou^†^, and Changxi Yang***

State Key Laboratory of Precision Measurement Technology and Instruments, Department of Precision Instruments, Tsinghua University, Beijing 100084, China

*cxyang@tsinghua.edu.cn

**^†^**these authors contributed equally to this work


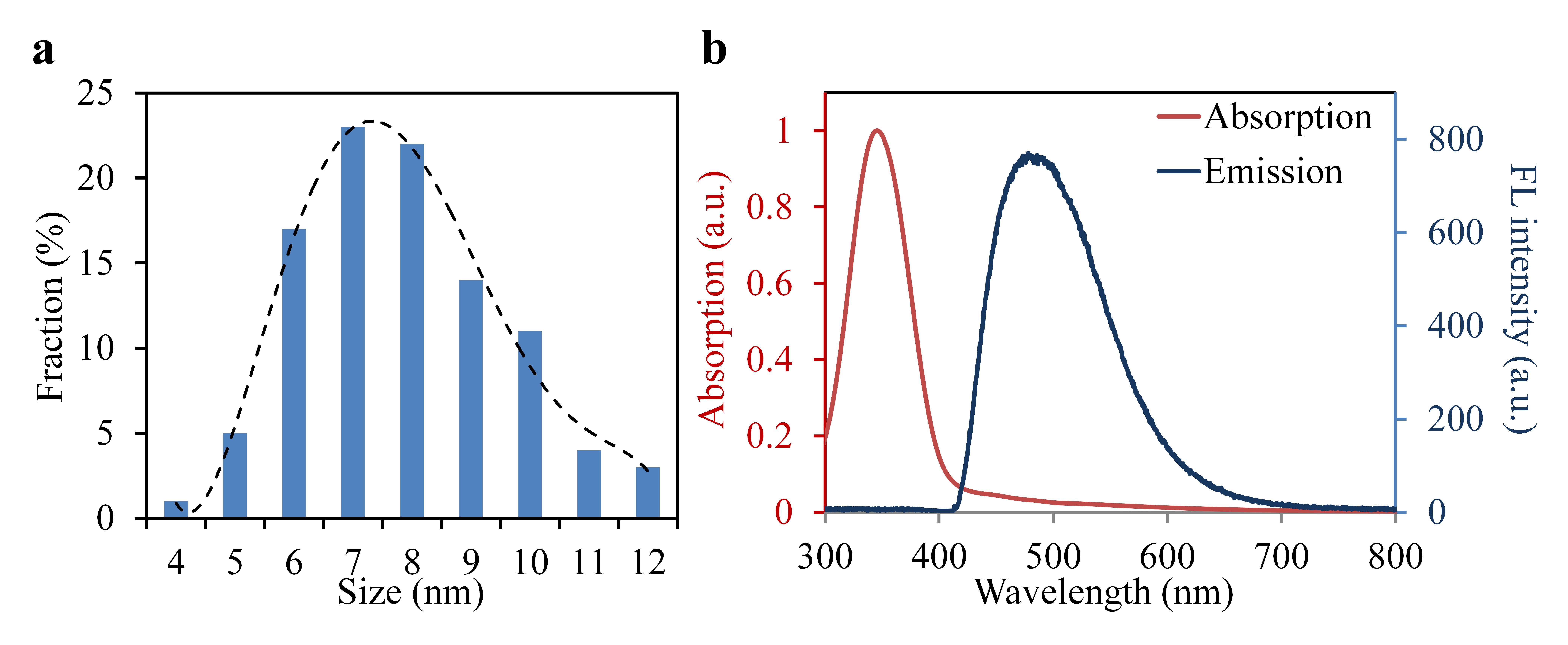


**Figure S1. a,** Size distribution of the CDs. **b**, Fluorescence emission and absorption spectra of the CDs in deionized water. Fluorescence spectrum was measured when the CDs were excited by laser at 405 nm.


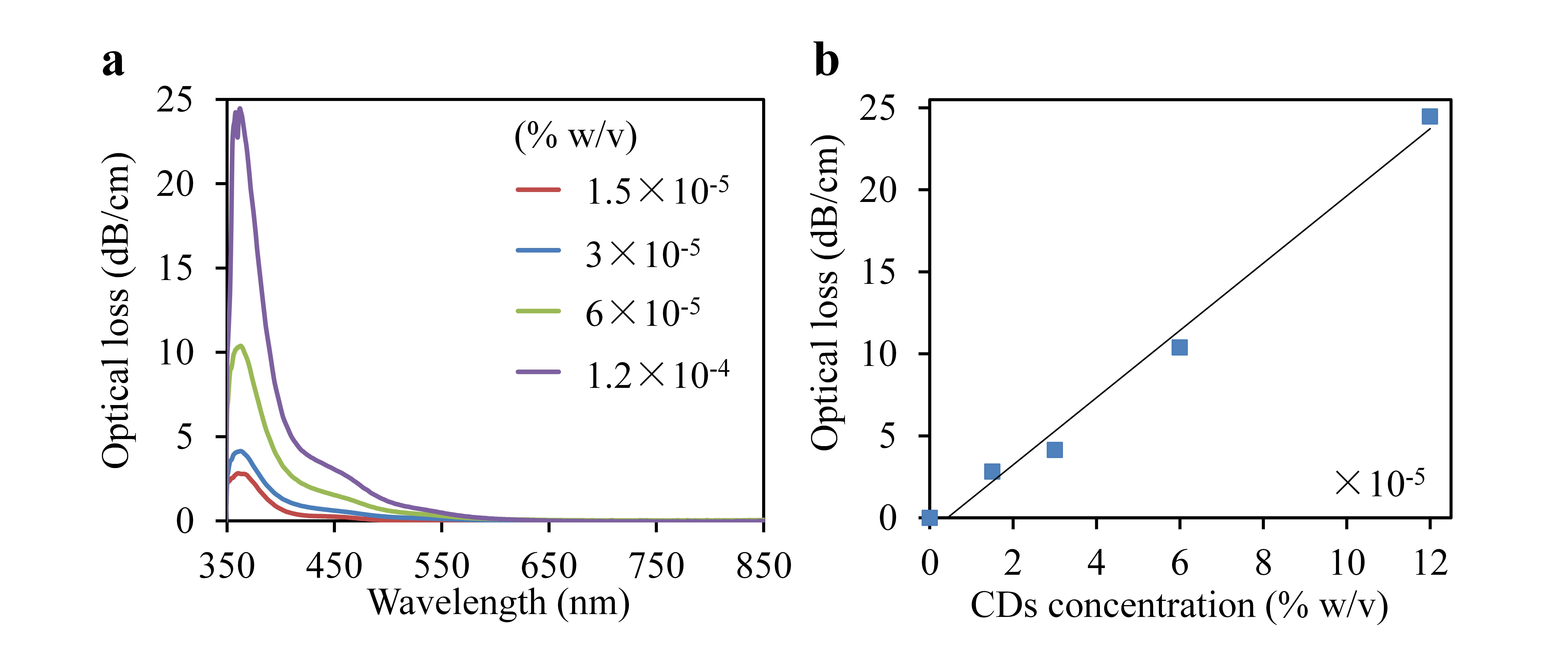


**Figure S2. a,** Optical loss spectra contributed by absorption of the CDs, obtained by subtracting loss spectra of PEGDA hydrogel from those of CDs-PEGDA nanocomposites. **b**, Optical attenuation at peak wavelength.

**
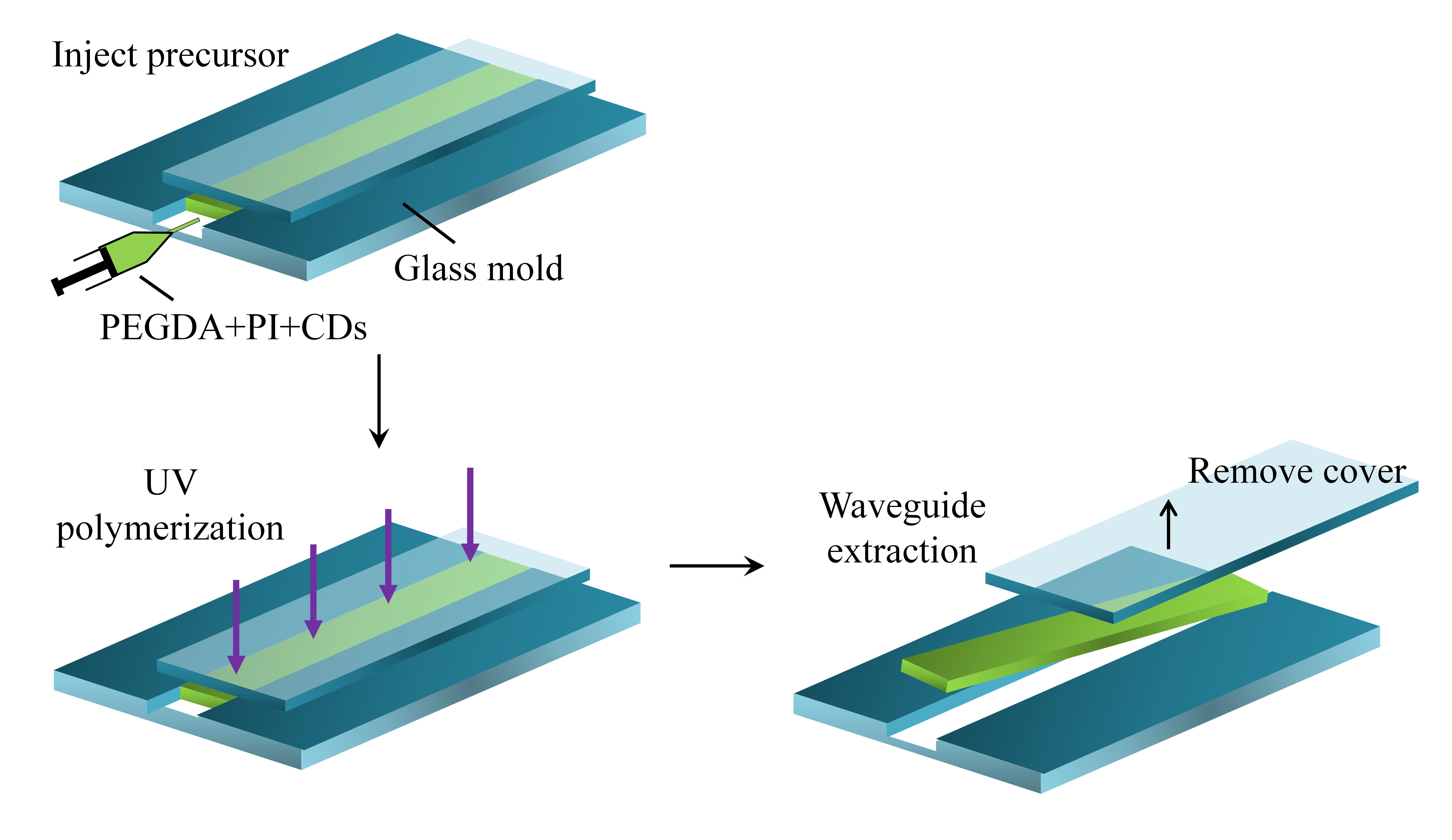
**

**Figure S3.** Fabrication steps. Slab waveguide was fabricated by injecting the CDs-PEGDA precursor in a rectangular mold with a syringe. After polymerization under UV irradiation, the waveguide was taken out by removing the cover slide.

**
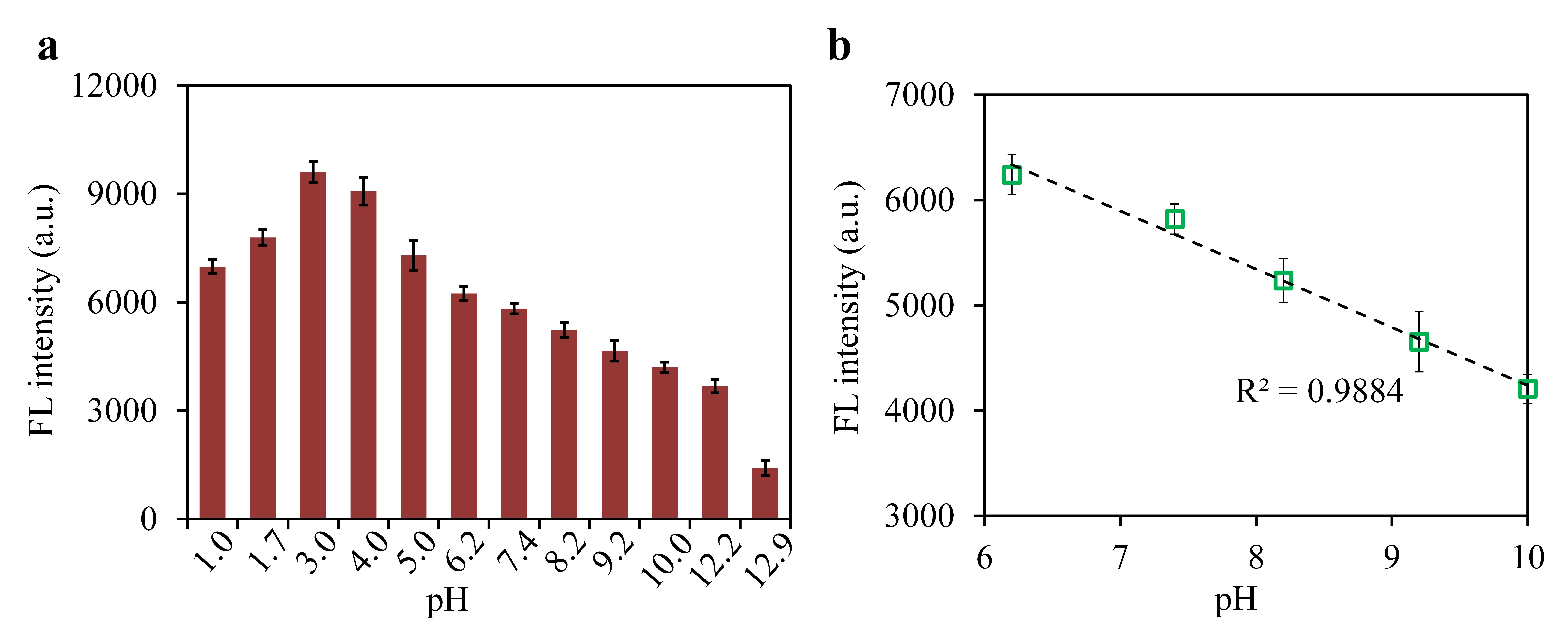
**

**Figure S4.** **a**, Dependence of fluorescence intensity on pH. **b**, Linear response of fluorescence intensity to pH in range of 6.2-10.0. Error bars, standard deviations (n=3).


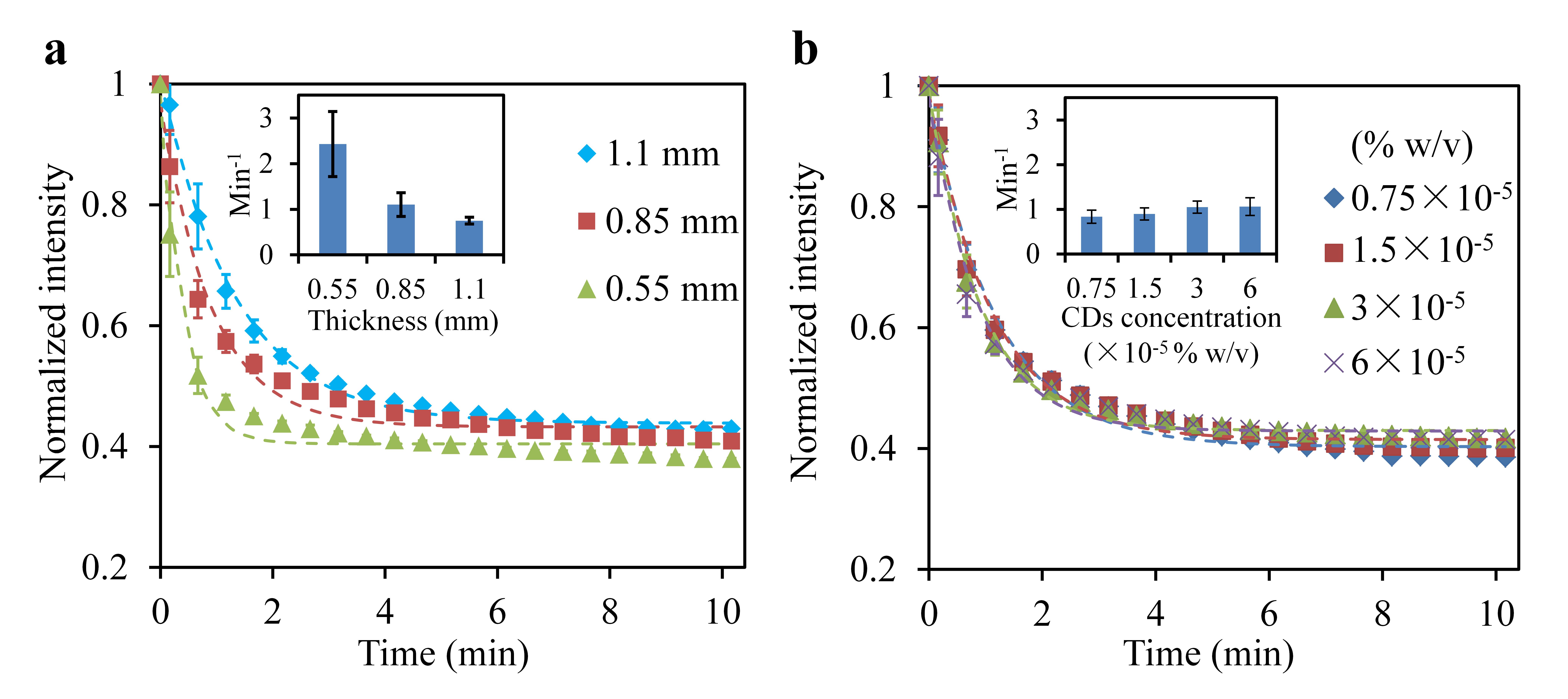


**Figure S5.** Time responses of waveguides to Hg^2+^ (2.5 μM) at various waveguide thickness (**a**) and CDs concentration (**b**). Dot lines, curve fits with the exponential decay equation. The inset in **a** and **b** shows the fitted decay constant. Error bars, standard deviations (n=3).
